# Phytochemical Profiling, Antimicrobial and α-Glucosidase Inhibitory Potential of Phenolic-Enriched Extracts of the Aerial Parts from *Echium humile* Desf.: In Vitro Combined with In Silico Approach

**DOI:** 10.3390/plants11091131

**Published:** 2022-04-21

**Authors:** Kaïss Aouadi, Hafedh Hajlaoui, Soumaya Arraouadi, Siwar Ghannay, Mejdi Snoussi, Adel Kadri

**Affiliations:** 1 Department of Chemistry, College of Science, Qassim University, Buraidah 51452, Saudi Arabia; k.aouadi@qu.edu.sa (K.A.); s.ghannay@qu.edu.sa (S.G.); 2 Department of Chemistry, Faculty of Sciences of Monastir, University of Monastir, Avenue of the Environment, Monastir 5019, Tunisia; 3 Research Unit Valorization and Optimization of Resource Exploitation (UR16ES04), Faculty of Science and Technology of Sidi Bouzid, Campus University Agricultural City, University of Kairouan, Sidi Bouzid 9100, Tunisia; bio.hafedh@gmail.com; 4 Regional Center of Agricultural Research (CRRA) Sidi Bouzid, Gafsa Road Km 6, PB 357, Sidi Bouzid 9100, Tunisia; bio.soumaya@gmail.com; 5 Research Laboratory, Valorization of Non-Conventional Waters, University of Carthage, Road Hedi EL Karray, El Menzah IV, PB 10, Ariana 2080, Tunisia; 6 Department of Biology, College of Science, Hail University, Ha’il 2440, Saudi Arabia; snmejdi@gmail.com; 7 Laboratory of Genetic, Biodiversity and Valorization of Bioressources, Higher Institute of Bio-Technology of Monastir, University of Monastir, Avenue Taher Hadded, B.P. 74, Monastir 5000, Tunisia; 8 Department of Chemistry, Faculty of Science of Sfax, University of Sfax, B.P. 1171, Sfax 3000, Tunisia; 9 Faculty of Science and Arts in Baljurashi, Albaha University, Albaha 65527, Saudi Arabia

**Keywords:** antidiabetic, antimicrobial, *Echium humile*, extracts, HPLC–MS analysis, molecular docking

## Abstract

The current study aimed to evaluate the naturally occurring antimicrobial and antidiabetic potential of various *Echium humile* (*E. humile*) solvent extracts (hexane, dichloromethane, ethyl acetate, methanol and aqueous). The bioactive compounds were identified using HPLC–MS, revealing the presence of sixteen phytochemical compounds, with the most abundant being *p*-coumaric acid, followed by 4,5-di-*O*-caffeoylquinic acid, trans-ferulic acid and acacetin. Furthermore, *E. humile* extracts showed marked antimicrobial properties against human pathogen strains, with MIC values for the most relevant extracts (methanol and ethyl acetate) ranging from 0.19 to 6.25 mg/mL and 0.39 to 12.50 mg/mL, respectively. Likewise, methanol was found to be bactericidal towards *S. aureus*, *B. cereus* and *M. luteus*, fungicidal against *P. catenulatum* and *F. oxysporum* and have a bacteriostatic/fungicidal effect for the other strains. In addition, the *E. humile* methanolic extract had the greatest α-glucosidase inhibitory effect (IC_50_ = 0.06 ± 0.29 mg/mL), which is higher than the standard drug, acarbose (IC_50_ = 0.80 ± 1.81 mg/mL) and the aqueous extract (IC_50_ = 0.70 ± 0.67 mg/mL). A correlation study between the major phytochemicals and the evaluated activities was investigated. Docking studies evidenced that most of the identified phenolic compounds showed strong interactions into the binding sites of *S. aureus* tyrosyl-tRNA synthetase and human lysosomal acid-α-glucosidase, confirming their suitable inhibitory effect. In summary, these results may provide rational support to explore the clinical efficacy of *E. humile* and its secondary metabolites in the treatment of dual diabetes and infections.

## 1. Introduction

Diabetes mellitus (DM) is a degenerative chronic metabolic disorder associated with various types of infections, with the most frequent being type 2 diabetes mellitus (T2DM). T2DM is an autoimmune and preventable disease, characterized by impaired/absence of insulin signaling, and is associated with various degrees of pancreatic β-cell failure in hepatic, adipose and muscle tissues, as well as reduced insulin sensitivity, leading to endocrine abnormalities and persistent hyperglycemia [1,2]. According to the IDF Middle East and North Africa (MENA) estimation, the prevalence of diabetes in the Kingdom of Saudi Arabia reached 18.3% in June 2020, including 4,275,200 total cases of diabetes in adults. The Saudi Scientific Diabetes Society indicates that more than 52% of patients with *DM2* die of cardiovascular causes [3]. On the other hand, pathogenic infections have threatened human health for many years due to the excessive abuse and misuse of antimicrobial agents [4]. Diabetic patients are prone to antimicrobial-resistant bacterial pathogens, which may be associated with a higher risk of mortality [5]. The risk to be diabetic increases dramatically (two-fold) for patients suffering from infectious disease (naturally taking more antibiotics), which alters and weakens their immunity [6]. In addition, some pathogens, such as *Staphylococcus* spp., *Salmonella enterica* and *Mycobacterium tuberculosis*, are associated, and *Klebsiella* spp. is linked to DM [4]. Akash et al. [6] showed the abundance of some bacterial strains identified in diabetic patients such as *Enterococcus* spp. (4%), *Staphylococcus* spp. (5%), *Klebsiella* spp. (6%) and *E. coli* (71%). More *Enterococcus* spp. than *Klebsiella* spp. and *Staphylococcus* spp. was found in patients without DM. Thus, searching for preventive and therapeutic strategies has become urgent in order to avoid the undesirable adverse effects caused by current synthetic drugs such as voglibose, miglitol and acarbose [7,8]. These drugs serve as oral hypoglycemic medications to inhibit α-amylase and α-glucosidase and, therefore, decrease the postprandial blood sugar levels in borderline patients [9,10,11]. α-Glucosidase is released from intestine cells and hydrolyzes oligosaccharides and polysaccharides to the small monosaccharides. Thus, α-glucosidase inhibitors play a pivotal role in controlling T2DM [12]. α-Glucosidase is capable of ameliorating hyperglycemia, especially postprandial hyperglycemia over α-amylase inhibitors, and its inhibition facilitates the maintenance of circulating glucose levels by decreasing the rate of blood sugar absorption [13]. As aforementioned, the high emergence of multidrug-resistant bacterial and fungal strains, as well as the increased attention to inhibiting the digestive enzymes linked to T2DM, has encouraged researchers to pay more interest to plant-based phytochemicals, although optimal treatment effects are yet to be achieved [14,15]. Therefore, medicinal plant-based therapies have gained great attention, given their rich constituents and metabolites [16,17]. They have been used to cure infections and different types of diseases by offering attractive, effective and holistic drug action without side effects [18,19,20]. Additionally, they can avoid excessive free radicals and exert many positive health benefits due to their richness in phenolic compounds acting as antioxidant, antimicrobial and antidiabetic agents, among others [21,22,23,24,25,26].

*Echium humile* Desf. (syn., *Echium pycnanthum* ssp.) is a wild plant species belonging to the Boraginaceae family. It is a small hispid biennial to perennial herb commonly found in dry and desert places, well recognized as a traditional remedy and largely used to treat liver disease, digestive ailments and hepatitis [27].

In the continuing effort to develop an effective therapeutic solution against target proteins [28,29,30,31], and due to the increasing global prevalence of diabetes associated with high mortality from infection in diabetic patients, the present work aimed to assess, for the first time, the antimicrobial and antidiabetic inhibitory effect of *E. humile* aerial parts using different extraction solvents (hexane, dichloromethane, ethyl acetate, methanol and aqueous). Furthermore, the phytochemical compounds of the most active extract(s) were investigated by HPLC–MS analysis, correlated with the tested activities and their possible binding interaction at the active site of TyrRS for *S. aureus* tyrosyl-tRNA synthetase (PDB ID: 1JIJ) and human lysosomal acid-α-glucosidase (PDB ID: 5NN8) in order to describe the “best-fit” orientation of a ligand that binds to a particular protein of interest.

## 2. Results

### 2.1. Biological Properties

#### 2.1.1. Antimicrobial Activity

Plant-based phytochemicals offer attractive, effective and holistic drug action against microorganisms with minimal side effects. The antimicrobial susceptibility assays were initially performed to determine the inhibitory effect of the various extracts. Data revealed that all studied extracts showed significant antimicrobial activity against the tested strains and that the activity varied from strains (Table 1). Except for *S. aureus*, *E. coli* and those from fungal strains, the aqueous extract was found to be active towards all tested strains, with recorded IZDs in the range of 9.00 ± 0.00–15.00 ± 0.00 mm. Likewise, *M. luteus* and *B. subtilis* exhibited high resistance to hexane and dichloromethane extracts. In contrast, the methanolic extract inhibited the growth of *E. faecalis* (15.50 ± 1.00 mm) and *F. oxysporum* (20.00 ± 0.00 mm), respectively, with values not significantly different (*p* > 0.05) from those of the standards, chloramphenicol and cycloheximide. The statistical analysis shows significant differences (*p* < 0.05) in the most susceptible strains, i.e., *M. luteus* (hexane and aqueous extracts), *E. coli* (dichloromethane extract), *S. aureus*, *M. luteus* and *F. oxysporum* (ethyl acetate extract), *E. faecalis* and *F. oxysporum* (methanolic extract) and others.

In addition, all extracts were examined for their ability to inhibit growth (MIC) or cause death (MBC/MIC and MFC/MIC) of strains (Table 2). Results outlined that MIC values for the most relevant methanolic and ethyl acetate extracts ranged from 0.19 to 6.25 mg/mL and 0.39 to 12.5 mg/mL, respectively. Methanol was found to be bactericidal (MBC/MIC = 2–4) against *S. aureus*, *B. cereus* and *M. luteus*, fungicidal (MBC/MFC = 2–4) against *P. catenulatum* and *F. oxysporum* and have a bacteriostatic or fungicidal effect for the other strains. Hexane and dichloromethane extracts seemed to exert a bactericidal effect towards all strains (except *B. cereus*); however, ethyl acetate was found to be bactericidal only against *M. luteus*, *K. pneumoniae* and *S. Enteritidis* (MBC/MIC = 2–4) and fungicidal against *P. catenulatum* and *F. oxysporum* (MBC/MFC = 4), while aqueous extract had a bacteriostatic effect against only *M. luteus* and *E. faecalis* (MBC/MIC = 16–32).

#### 2.1.2. Antidiabetic Activity

T2DM is a metabolic disorder of protein, fat and carbohydrate metabolism characterized by persistent hyperglycemia with serious complications. α-Glucosidase is one of the enzymes that inhibit the digestion of carbohydrates into glucose and promotes glucose conversion; therefore, its inhibition has been used for the treatment of T2DM. Inhibition of the α-glucosidase enzyme can help in delaying the digestion of carbohydrates, thereby reducing the levels of glucose in the blood. In this study, an antidiabetic bioassay was carried out for the first time to test the potentiality of *E. humile* extracts as an α-glucosidase inhibitor. As shown, among the different extracts, methanolic extract exhibited maximum inhibitory activity (IC_50_ = 0.06 ± 0.29 mg/mL), which is about 12-fold higher than the commercial standard, acarbose (IC_50_ = 0.80 ± 1.81 mg/mL) and the aqueous extract (IC_50_ = 0.70 ± 0.67 mg/mL), which were non-significantly different (*p* > 0.05), while ethyl acetate displayed moderate activity, and hexane and dichloromethane were inactive (Table 3).

### 2.2. HPLC–MS Analysis

The phenolic compounds of the most active extract (methanol extract) were tentatively identified by HPLC–MS analysis. As shown in Table 4, a total of sixteen components with their identities, retention times (Rts), pseudomolecular ions [M−H]^-^ and levels were found with *p*-coumaric acid (1335.48 µg/g)*,* 4,5-di-*O*-caffeoylquinic acid (319.373 µg/g), *trans*-ferulic acid (125.522 µg/g) and acacetin (107.462 µg/g) obtained with a [M−H]^−^ ion at *m/z* 163, 515, 193 and 283, respectively, which were found to be the major phenolics in methanol *E. humile* extract. Additionally, medium levels of salvianolic acid (47.832 µg/g), cirsiliol (41.623 µg/g), rosmarinic acid (41.154 µg/g), (+)-catechin (39.286 µg/g), epicatechin (26.868 µg/g) and 1,3-di-*O*-caffeoylquinic acid (22.367 µg/g) with a pseudomolecular ion at *m/z* 717, 329, 359, 289, 289 and 515, and lower amounts of caffeic acid (9.673 µg/g), 3,4-di-*O*-caffeoylquinic acid (6.7645 µg/g), protocatechuic acid (4.204 µg/g) hyperoside (quercetin 3-*O*-galactoside) (2.992 µg/g), apigenin 7-*O*-glucoside (2.481µg/g) and apigenin (0.973µg/g), were detected as well.

### 2.3. Computational Study

#### 2.3.1. Binding Energies

**Figure 1:**
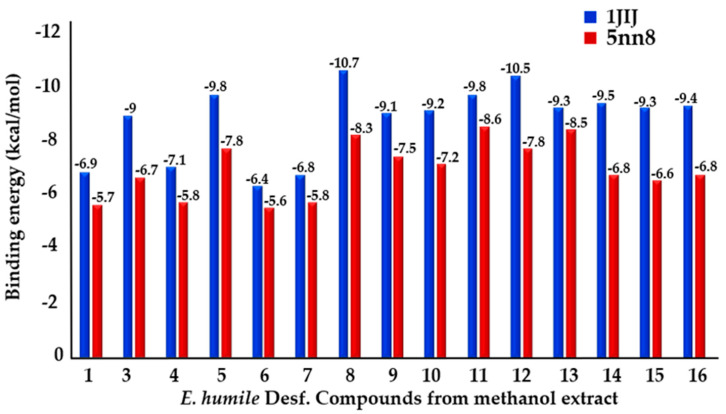
Binding energies of *E. humile* Desf. compounds complexed with TyrRS from *S. aureus* (PDB: 1JIJ) and human lysosomal acid-α-glucosidase (PDB: 5NN8) enzymes.

In order to provide insight into which of the identified phytocompound(s) may be responsible for dual antimicrobial and antidiabetic activities, a molecular docking study was carried out on the following different protein structures: TyrRS from *S. aureus* (PDB: 1JIJ) and human lysosomal acid-α-glucosidase (PDB: 5NN8) enzymes, to modulate the inhibition potential of phytochemicals found in the most active extract against these target enzymes. The results revealed that all identified compounds exhibited good affinity (−10.7 kcal/mol ≤ phytocompounds-1JIJ ≤ −6.4 kcal/mol; −8.5 kcal/mol ≤ phytocompounds-5NN8 ≤ −5.6 kcal/mol) towards the investigated target enzymes (Figure 1).

#### 2.3.2. Receptor–Ligand Interaction Analysis

**Figure 2:**
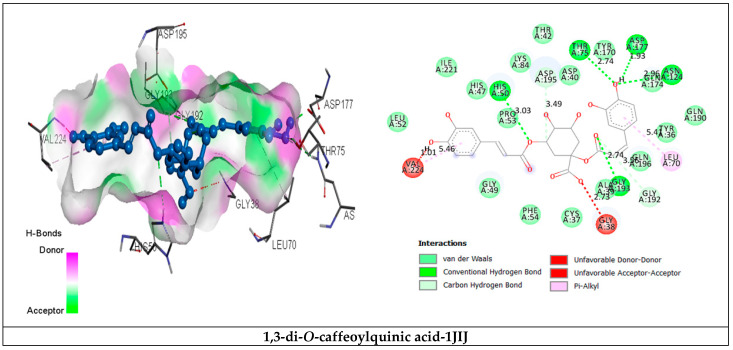

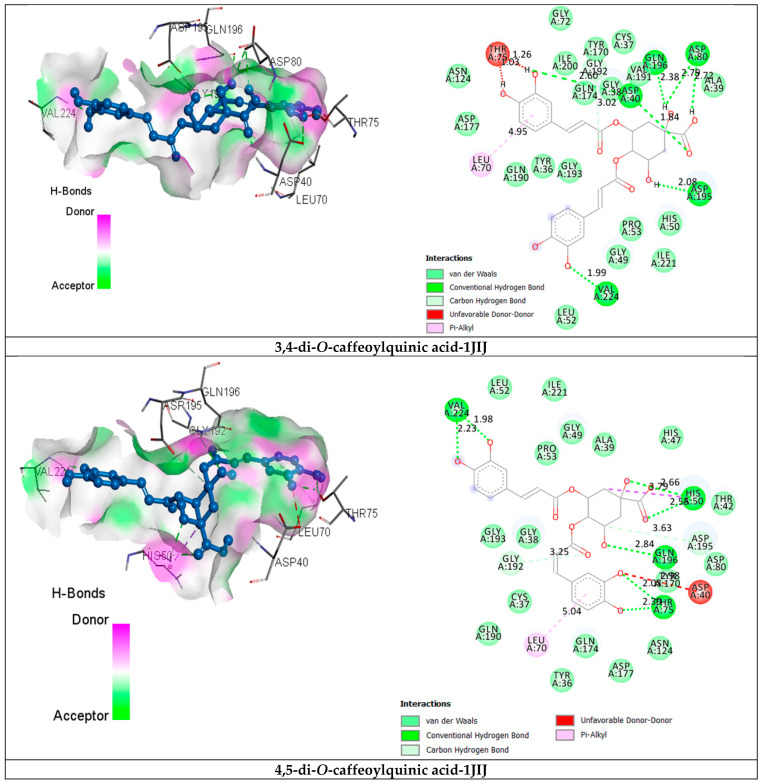

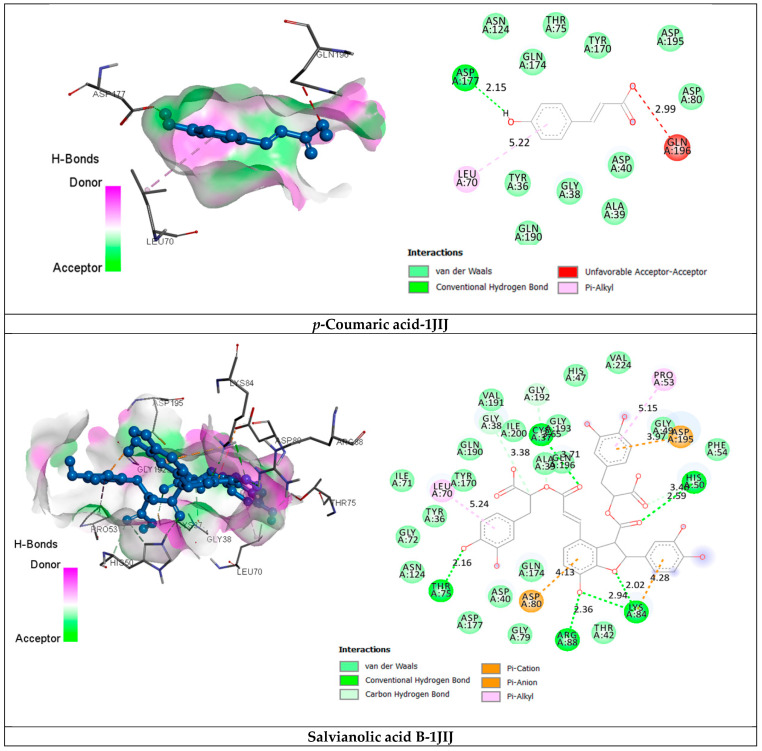
Interactions of tyrosyl-tRNA synthetase TyrRS from *S. aureus* (PDB: 1JIJ) with the selected major phytochemicals, with the top two having the lowest binding energies of *E. humile* methanolic extract.

Phytochemicals were selected based on their high concentration in the extract and/or lowest binding energy values in order to identify the key molecular scaffold responsible for the antimicrobial and antidiabetic activities. Their interaction types and attachment positions are shown in Table 5 and Figure 2. Considering the lowest binding energy, the phytochemicals 1,3-di-*O*-caffeoylquinic acid, 3,4-di-*O*-caffeoylquinic acid and 4,5-di-*O*-caffeoylquinic acid were the most potent scaffolds, with binding energies of −9.8 kcal/mol, −10.7 kcal/mol and -10.5 kcal/mol, respectively, bound in the active site and able to adopt several favorable H-bond contacts, as well as other interactions with the common catalytic residues involved in the ternary structure of TyrRS from *S. aureus* (Cys37, Gly38, Ala39, Asp40, His47, Gly49, His50, Leu70, Thr75, Gln174, Asp177, Gln190, Gly192, Asp195 and Pro222).

**Figure 3:**
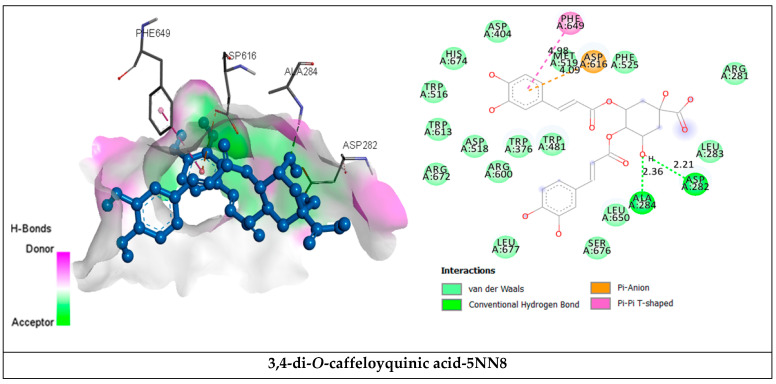

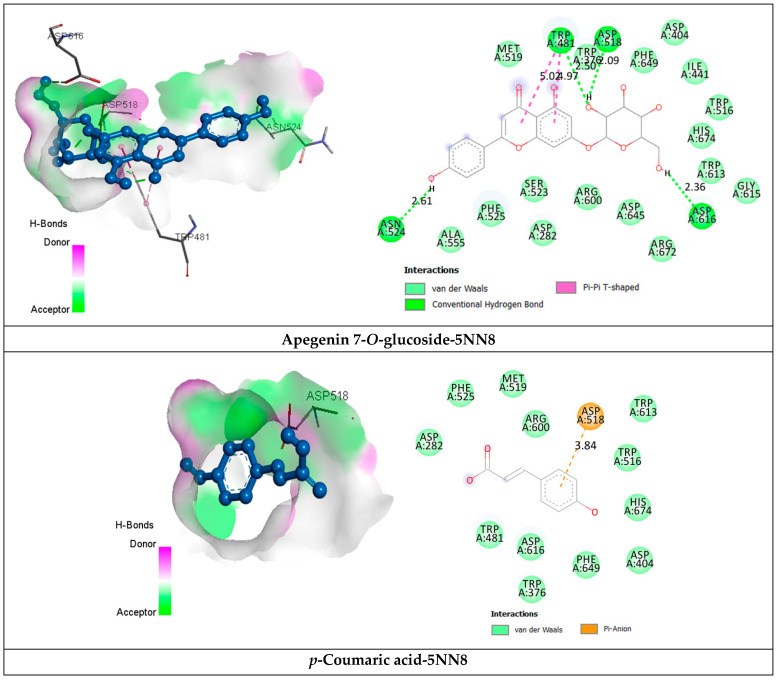

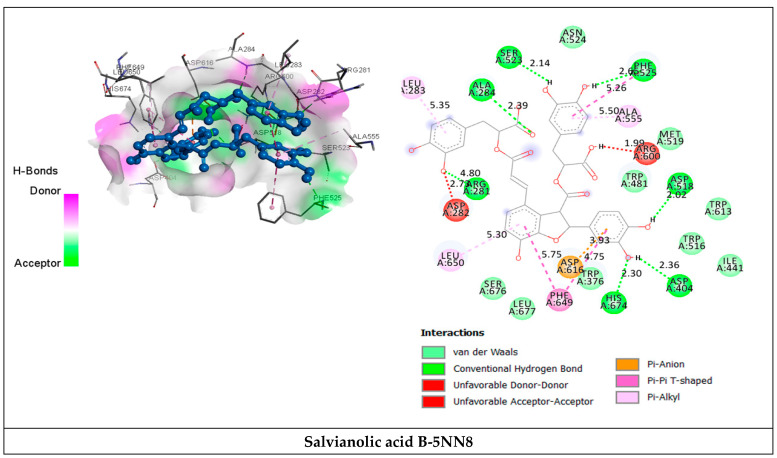
Interactions of human lysosomal acid-α-glucosidase (PDB: 5NN8) with the selected major phytochemicals, with the top two having the lowest binding energies of *E. humile* Desf. methanolic extract.

Interactions of selected phytocompounds inside α-glucosidase (Table 6 and Figure 3) show that there are two hydrogen bonds (Asp282 at 2.21 Å, Ala284 at 2.36 Å) formed with 3,4-di-*O*-caffeoylquinic acid, seven hydrogen bonds with salvianolic acid and enzyme residues Arg281 (4.80 Å), Ala284 (2.39 Å), Asp404 (2.36 Å), Asp518 (2.02 Å), Ser523 (2.14 Å), Phe525 (2.6 Å), His674 (2.30 Å) and four hydrogen bonds with apigenin 7-*O*-glucoside and Trp481 (2.50 Å), Asp518 (2.09 Å), Asn524 (2.61 Å), Asp616 (2.36 Å) amino acids with, respectively, predicted binding energy of −8.3 kcal/mol, −8.6 kcal/mol and −8.5 kcal/mol.

## 3. Discussion

A structure–activity relationship (SAR) study was carried out to emphasize the importance of the discovery of new alternative drugs and highlight the different contributors to the powerful activity of various *E. humile* extracts. The biological activities of plant extracts depended on their identified phytocomponents. It is well known that ethanol, methanol and water are effective solvents, commonly used for the preliminary screening of plant extracts and withdrawal of most antimicrobial compounds containing aromatic and saturated organic compounds [31]. As recently verified by our team [26], *E. humile* extracts are very rich in polyphenols and flavonoid contents, which favor membrane disruption, followed by leakage of cellar components from the microbial membrane [32,33]. The authors also mentioned that methanolic extract exhibited a high amount of phenolic contents [26]. In this study, the high susceptibility of Gram-positive bacteria may be attributed to the composition difference in the bacteria’s cell wall, where the hydrophilic surface of the extra outer membrane in Gram-negative bacteria limits membrane permeability, acts as a barrier to the percolation of different antibacterial substations and prevents antimicrobial agents from penetrating the cell wall [31]. We mentioned that isolates of *E. faecalis* and *E. faecium* are among the most prevalent nosocomial pathogens worldwide displaying high resistance, particularly to penicillin/ampicillin and aminoglycosides (high-level resistance). Therefore, therapeutic alternatives to treat infections will require careful attention and remain essential for health care provision [34]. Based on the HPLC/MS phytochemical analysis in the docking study relationships, the main contributors to the strongest antimicrobial activity were identified as follows. As a secondary metabolite, the first major compound in the methanolic *E. humile* extract was *p*-coumaric acid (3-[4-hydroxyphenyl]-2-propenoic acid or 4-hydroxycinnamic acid), which has been well confirmed for its antioxidative properties. Indeed, Lou et al. [35] suggested that *p*-coumaric acid possesses dual mechanisms of bactericidal activity, disrupting bacterial cell membranes and binding to bacterial genomic DNA to inhibit cellular functions, ultimately leading to cell death. The same authors revealed in their study that the effectiveness of *p*-coumaric acid against *E. coli*, *S. typhimurium* and *S. dysenteriae* via the compound changes the permeability of the cell membrane in parallel with its capacity to bind to DNA-inhibiting cell function. Some authors have reported that the scavenging ability of *p*-coumaric acid is mainly attributed to its phenyl hydroxyl group (−OH) [36]. Amalan et al. [37] stated that the antidiabetic potential of *p*-coumaric acid provides a protective role in pancreatic β-cells of diabetic rats by reducing ROS-induced oxidative stress and improving antioxidant status. The authors demonstrated also that *p*-coumaric acid significantly decreases the blood glucose level and gluconeogenic enzymes such as glucose-6-phosphatase and fructose-1,6-bisphosphatase, but enhances the activities of hexokinase, glucose-6 phosphatase dehydrogenase and GSH via increasing the level of insulin. Additionally, it reduces the total cholesterol and triglycerides in both plasma and tissues, i.e., liver and kidney [37]. Recently, Shen et al. revealed that *p*-coumaric acid displayed potential therapeutic effects on hyperlipidemia [38]. Ferulic acid (4-hydroxy,3-methoxy cinnamic acid) is known to exert beneficial effects by scavenging free radicals and activating antioxidant enzymes, resulting in a reduction in cellular redox imbalance in a DM animal model. Georgiev et al. [39] reported that ferulic acid displayed potent inhibitory growth towards *S. aureus* and *S. pyogenes* 10535. Moreover, it has been mentioned that ferulic acid can alleviate the high-glucose-induced oxidative stress and decreased cell apoptosis in hepatocytes and cardiomyocytes, which are associated with the Keap1-Nrf2-ARE signaling pathway [40]. Ferulic acid possesses beneficial health effects against oxidative stress, hypertension and late-stage diabetes in obese rats, as well as significantly improving insulin sensitivity and lipid profiles and reducing elevated blood pressure [41,42]. Salvianolic acids, known as the most abundant water-soluble compounds extracted from *Salvia miltiorrhiza* (Danshen), are a powerful antioxidative agent that protects cells from peroxidation and free radical damage [43]. Salvianolic acid B can protect pancreatic beta-cells against cytotoxicity and prevent high-glucose-induced apoptosis [10]. Raoufi et al. [10] suggested that treatment of diabetic rats with salvianolic B induces antidiabetic effects by protecting pancreatic β-256 cells against chemical insult and ameliorates the insulin secretory function of β-cells. Rosmarinic phenylpropanoid acid is an ester of caffeic acid. 3,4-Dihydroxyphenyl lactic acid is known as the strongest antioxidant of all hydroxycinnamic acid derivatives, and its antibacterial activity against *S. aureus* and the clinical isolate of methicillin-resistant *Staphylococcus aureus* (MRSA) has been shown [44,45]. Rosmarinic acid has also been shown to exert antidiabetic effects [46,47].

Acacetin (5,7-dihydroxy-4-methoxyflavone) is a naturally occurring flavonoid (synonym linarigenin) that displayed moderate antimicrobial activities against several Gram-positive bacteria (*Actinomyces naeslundii, Actinomyces Israelii, Streptococcus mutans*), as well as against a variety of Gram-negative bacteria (*Prevotella intermedia, Porphyromonas gingivalis, Aggregatibacter actinomycete mcomitans*) [48]. As a bioactive constituent of *Combretum vendae* (Combretaceae), acacetin exhibited strong activity against *Staphylococcus aureus, Enterococcus faecalis, Escherichia coli* and *Pseudomonas aeruginosa* [49]. It has been shown to exhibit antihyperglycemic activity in streptozotocin-induced diabetic mice at oral doses of 3 and 31.6 mg/kg by decreasing blood glucose levels in healthy and hyperglycemic mice when compared to an untreated group [50]. Acacetin displayed stable binding to the active site of aldose reductase, a primary mediator of diabetes-induced oxidative stress in retinopathy [51].

The obtained data are in good agreement with previous work, justifying the potential increased inhibitory effect against the α-glucosidase enzyme with increasing polarity of plant extracts [52]. Jesus et al. [53] reported that the infusion extract of *Prunus avium* vegetal stems parts is the most active. Additionally, methanolic *Cornus capitata* Wall. extract exhibited maximum inhibitory activity with 98.37% inhibition (IC_50_ = 12.5 μg/mL), better than that shown by acarbose. Likewise, *Arbutus pavarii* has been reported to exhibit potent α-glucosidase inhibitory activities [54].

The findings confirm the powerful use of *E. humile* extract to treat dual diabetes and infections.

## 4. Materials and Methods

### 4.1. Plant Material and Extraction

The aerial parts of *E. humile* were collected during the flowering stage from the region of Douz (Kebilli, South of Tunisia). Extracts were prepared following the same protocol as described by Aouadi et al. [26]. Briefly, extracts were obtained by maceration of 200 g of powder plant material with 600 mL of each solvent (hexane, dichloromethane, ethyl acetate, methanol and aqueous) for 72 h, filtered with Whatman filter paper and then collected and concentrated using a rotary evaporator at 35–55 °C. After that, the obtained extracts were kept in the dark at 4 °C until further use.

### 4.2. Antimicrobial Activity

The microorganisms tested in this study belonged to 18 reference bacterial strains and 5 fungal strains, which are presented in Table 3 and Table 4, respectively. Experimental details are the same as Hajlaoui et al. [13].

### 4.3. α-Glucosidase Inhibitory Assay

The α-glucosidase assay of the tested extract was conducted according to the previous protocol with slight modification [55]. The enzyme inhibition rate expressed as a percentage of inhibition was calculated using the following formula:

Percentage inhibitory activity (%) = (1 − A/B) × 100

where A is the absorbance in the presence of the test substance, and B is the absorbance in the presence of phosphate buffer (control).

### 4.4. HPLC–MS Analysis of Phenolic Compounds

The identification of polyphenolics was performed using the Shimadzu HPLC-MS 2020 system. Detailed experiments were the same as per the method reported by Hajlaoui et al. [26].

### 4.5. Molecular Docking Approach

Interactions between the selected identified bioactive phytocompounds and the receptor human peroxiredoxin 5 were assessed by in silico molecular docking, in order to explore the preferred orientation of the ligands in the binding site of receptors. We used the same protocol as described previously by our team [26]. The structures of the natural compounds were minimized as done previously [26].

### 4.6. Statistical Analysis

All experiments were performed in triplicates, and average values were calculated using the SPSS 25.0 statistical package for Windows. Differences in means were calculated using Duncan’s multiple range tests for means with a 95% confidence interval (*p* ≤ 0.05).

## 5. Conclusions

Our findings suggest that *E. humile* extracts showed significant antimicrobial potential against human pathogen strains, especially those of methanolic and ethyl acetate extracts. Additionally, *E.* *humile* methanolic extract exhibited a potent α-glucosidase inhibitory effect with respect to the commercial drug acarbose. The structure–activity relationship between the identified phytochemicals and the studied activities was assessed. Docking analysis indicated that the majority of compounds from the most active (methanolic) extract interact with the tested target enzymes through their preferential binding to the active site. Nevertheless, broad research should be conducted to isolate the main compounds from *E. humile* methanolic extract and explore their clinical efficacy in the treatment of diabetes and infections.

## Figures and Tables

**Table 1:** Antibacterial and antifungal activities of *E. humile* extracts.

Strains	IZD (mm)						
	Extracts	Hexane	Dichloromethane	Ethyl Acetate	Methanol	Aqueous	Chloramphenicol
Gram-positive bacteria							
*Staphylococcus aureus*		–	11.50 ± 1.00 ^cdC^	14.00 ± 2.00 ^abB^	10.00 ± 0.00 ^dCD^	9.00 ± 0.00 ^cD^	16.50 ± 1.00 ^dA^
*Enterococcus faecalis*		10.50 ± 1.00 ^cC^	13.00 ± 1.00 ^bcB^	12.50 ± 1.00 ^bcBC^	15.50 ± 1.00 ^aA^	10.50 ± 1.00 ^bC^	12.00 ± 1.00 ^eBC^
*Bacillus cereus*		14.00 ± 1.00 ^bB^	14.00 ± 1.00 ^bB^	12.00±1.00 ^cC^	14.00±1.00 ^bB^	11.50 ±1.00 ^bC^	26.00 ± 1.00 ^aA^
*Bacillus subtilis*		11.50 ± 1.00 ^cC^	14.50 ± 1.00 ^bB^	15.00 ± 1.00 ^aB^	12.50 ± 1.00 ^cC^	–	24.00 ± 0.00 ^bA^
*Micrococcus luteus*		18.50 ± 0.86 ^aA^	_	14.5 ± 1.00 ^aB^	14.00 ± 0.00 ^bB^	15.00 ± 0.00 ^aB^	20.00 ± 2.00 ^cA^
Gram-negative bacteria							
*Escherichia coli*		10.33 ± 0.76 ^cC^	18.00 ± 1.00 ^aB^	8.00 ± 0.00 ^eD^	–	–	23.50 ± 1.50 ^bA^
*Klebsiella pneumoniae*		11.00 ± 0.00 ^c^	11.00 ± 0.00 ^dB^	11.50 ± 1.50 ^cdB^	–	10.50±0.50 ^bB^	22.00 ± 1.00 ^bA^
*Salmonella Enteritidis*		8.00 ± 0.00 ^dE^	11.50 ± 0.50 ^cdB^	10.00 ± 0.00 ^dC^	11.50 ± 0.50 ^cB^	9.00 ± 0.00 ^cD^	16.00 ± 0.00 ^dA^
Fungal strains							Cycloheximide
*Fusarium* sp.		–	–	11.5 ± 0.71 ^bC^	14.5 ± 0.71 ^bB^	–	18.00 ± 1.50 ^abA^
*Pythium catenulatum*		–	–	9.00 ± 0.00 ^cB^	10.50 ± 0.71 ^cB^	–	17.50 ± 1.50 ^bA^
*Fusarium oxysporum*		–	–	13.00 ± 1.00 ^aB^	20.00 ± 0.00 ^aA^	–	20.00 ± 2.00 ^aA^

Values are mean ± standard deviation of three separate experiments. Diameter of inhibition zones of extract including diameter of well is 6 mm; –: no inhibition; a,b,c,d,e,f,g,h,A,B,C,D,E: each value represents the average of 3 repetitions. Small letters are used to compare each extract means between different strains, while capital letters are used to compare means between extracts for the same strain.

**Table 2:** Determination of MIC (MBC or MFC) in mg/mL and {MBC/MIC or MFC/MIC} of *E. humile* extracts.

Strains	Hexane	Dichloromethane	Ethyl Acetate	Methanol	Aqueous
Gram-positive bacteria					
*Staphylococcus aureus*	–	12.5 (25) {2}	1.56 (12.5) {8}	3.12 (6.25) {2}	3.12 (6.25) {2}
*Enterococcus faecalis*	6.25 (12.5) {2}	12.5 (50) {4}	1.56 (12.5) {8}	0.19 (3.12) {16}	0.19 (6.25) {32}
*Bacillus cereus*	0.39 (6.25) {16}	6.25 (25) {4}	12.5 (100) {8}	6.25 (12.5) {2}	–
*Bacillus subtilis*	6.25 (25) {4}	50 (100) {2}	1.56 (12.5) {8}	0.39 (6.25) {16}	0.19 (3.12) {16}
*Micrococcus luteus*	12.5 (50) {4}	–	1.56 (6.25) {4}	6.25 (25) {4}	3.12 (6.25) {2}
Gram-negative bacteria					
*Escherichia coli*	3.12 (12.5) {4}	1.56 (6.25) {4}	1.56 (12.5) {8}	–	–
*Klebsiella pneumoniae*	50 (100) {2}	25 (50) {2}	12.5 (25) {2}	–	3.12 (6.25) {2}
*Salmonella Enteritidis*	1.56 (3.12) {2}	3.12 (12.5) {4}	1.56 (3.12) {2}	0.39 (3.12) {8}	1.56 (6.25) {4}
Fungal strains					
*Fusarium* sp.	–	–	0.39 (3.12) {8}	0.39 (3.12) {8}	–
*Pythium catenulatum*	_	_	1.56 (6.25) {4}	0.195 (0.78) {4}	–
*Fusarium oxysporum*	–	–	0.39 (1.56) {4}	3.12 (6.25) {2}	–

**Table 3:** IC_50_ values of various extracts of *E. humile* against α-glucosidase.

Extracts	IC_50_ (mg/mL)
Hexane	–
Dichloromethane	–
Ethyl acetate	11.17 ± 0.62 ^a^
Methanol	0.06 ± 0.29 ^c^
Aqueous	0.80 ± 1.81 ^b^
Acarbose	0.70 ± 0.67 ^b^

Means followed by the same letters are not significantly different at *p* = 0.05 based on Duncan’s multiple range test.

**Table 4:** HPLC–MS analysis of *E. humile* methanolic extract from aerial parts.

Peak	Retention Time (min)	MS [M-H]^-^m/z	Compounds	Concentration (µg/g)
1	7.385	153.00	Protocatechuic acid	4.204 ± 0.06
2	9.189	289.00	(+)-Catechin	39.286 ± 2.46
3	13.795	289.00	Epicatechin	26.868 ± 0.82
4	12.993	179.00	Caffeic acid	9.673 ± 0.1
5	14.960	515.00	1,3-di-*O*-caffeoylquinic acid	22.367 ± 0.15
6	17.087	163.00	*p*-Coumaric acid	1335.48 ± 5.22
7	18.744	193.00	*Trans*-Ferulic acid	125.522 ± 10.3
8	21.779	515.00	3,4-di-*O*-caffeoylquinic acid	6.7645 ± 0.41
9	22.209	359.00	Rosmarinic acid	41.154 ± 0.12
10	22.910	463.00	Hyperoside	2.992 ± 0.01
11	23.754	717.00	Salvianolic acid B	47.832 ± 1.51
12	23.754	515.00	4,5-di-*O*-caffeoylquinic acid	319.373 ± 0.41
13	24.302	431.00	Apegenin 7-*O*-glucoside	2.481 ± 0.48
14	23.451	329.00	Cirsiliol	41.623 ± 0.19
15	31.852	269.00	Apegenin	0.973 ± 0.25
16	37.061	283.00	Acacetin	107.462 ± 17.4

**Table 5:** Major phytochemicals, with the top two having the lowest binding energies with their interaction residues with TyrRS from *S. aureus* (PDB: 1JIJ).

Compounds	Interactions Type	Interacting Residues (1JIJ)	Binding Energy (kcal/mol)
1,3-di-*O*-caffeoylquinic acid	van der Waals H bond C-H bond Unfavorable Donor-Donor/ Acceptor-Acceptor Pi-Alkyl	Tyr36, Cys37, Ala39, Thr42, His47, Gly49, Leu52, Pro53, Phe54, Lys84, Tyr170, Gln174, Gln190, Gln196, Ile221. His50 (3.03), Thr75 (2.74), Asp177 (1.93), Asn124 (2.96), Gly193 (2.74). Gly192 (3.56), Asp195 (3.38). Gly38 (2.77), Val224 (2.01). Leu70 (5.41), Val224 (5.46).	−9.8
3,4-di-*O*-caffeoylquinic acid	van der Waals H bond C-H bond Unfavorable Donor-Donor Pi-Alkyl	Tyr36, Cys37, Gly38, Ala39, Gly49, His50, Leu52, Pro53, Gly72, Asn124, Tyr170, Gln174, Asp177, Gln190, Val191, Gly193, Ile200, Ile221. Asp40 (1.84) (2.60), Asp80 (2.72) (2.79), Asp195 (2.08), Gln196 (2.38), Val224 (1.99). Gly192 (3.02). Thr75 (1.03) (1.26). Leu70 (4.95).	−10.7
4,5-di-*O*-caffeoylquinic acid	van der Waals H bond C-H bond Unfavorable Donor-Donor Pi-Alkyl	Tyr36, Cys37, Gly38, Ala39, Thr42, His47, Gly49, Leu52, Pro53, Asp80, Asn124, Tyr170, Gln174, Asp177, Gln190, Gly193, Ile221. His50 (2.55) (2.66), Thr75 (2.03) (2.39), Gln196 (2.84), Val224 (1.98) (2.23). Asp195 (3.36) Asp40 (2.98) Leu70 (5.04)	−10.5
*p*-Coumaric acid	van der Waals C-H bond Pi-Cation Pi-Alkyl	Tyr36, Gly38, Ala39, Asp40, Thr75, Asp80, Asn124, Tyr170, Gln174, Gln190, Asp195. Asp177 (2.15) Gln196 (2.99) Leu70 (5.22)	−6.4
Salvianolic Acid B	van der Waals H bond C-H bond Pi-Cation/Anion Pi-Alkyl	Tyr36, Ala39, Asp40, Thr42, His47, Gly49, Phe54, Ile71, Gly72, Gly79, Asn124, Tyr170, Gln174, Asp177, Gln190, Val191, Gly193, Gln196, Ile200, Val224. Cys37 (3.71), His50 (2.59), Thr75 (2.16), Lys84 (2.02) (2.94), Arg88 (2.36). Gly38 (3.38), His50 (3.46), Gly192 (3.65). Asp80 (4.13), Lys84 (4.28), Asp195 (3.97) Pro53 (5.15), Leu70 (5.24)	−9.8

**Table 6:** Major phytochemicals, with the top two having the lowest binding energies with their interaction residues with human lysosomal acid-α-glucosidase (PDB: 5NN8).

Compounds	Interactions Type	Interacting Residues (5NN8) (Å)	Binding Energy (kcal/mol)
3,4-di-*O*-caffeoylquinic acid	van der Waals H bond Pi-Anion Pi-Pi T shaped	Arg281, Leu283, Trp376, Asp404, Trp481, Asp518, Met519, Phe525, Trp516, Arg600, Trp613, Arg672, leu650, His674, Ser676, Leu677. Asp282(2.21), Ala284 (2.36). Asp616 (4.09) Phe649 (4.98)	−8.3
*p*-Coumaric acid	van der Waals Pi-Anion	Asp282, Trp376, Asp404, Trp481, Trp516, Met519, Phe525, Arg600, Trp613, Asp616, Phe649, His674. Asp518 (3.84).	−5.6
Salvianolic acid B	van der Waals H bond Unfavorable Donor-Donor/ Acceptor-Acceptor Pi-Anion Pi-Pi T shaped	Trp376, Ile441, Trp481, Trp516, Met519, Asn524, Trp613, Ser676, Leu677. Arg281 (4.80), Ala284 (2.39), Asp404 (2.36), Asp518 (2.02), Ser523 (2.14), Phe525 (2.6), His674 (2.30). Asp282 (2.73), Arg600 (1.99). Asp616 (3.93). Phe649 (4.75) (5.75).	−8.6
Apegenin 7-*O*-glucoside	van der Waals H bond Pi-Pi T shaped Pi-Alkyl	Asp282, Trp376, Ile441, Trp516, Met519, Ser523, Phe525, Ala555, Arg600, Trp613, Gly615, Asp645, Phe649, Arg672, His674. Trp481 (2.50), Asp518 (2.09), Asn524 (2.61), Asp616 (2.36). Trp481 (4.97) (5.02). Leu283 (5.35), Ala555 (5.50), Leu650 (5.30).	−8.5

## Data Availability

The data generated and analyzed during this study are included in this article.
